# Antibody induction and immune response in nasal cavity by third dose of SARS-CoV-2 mRNA vaccination

**DOI:** 10.1186/s12985-023-02113-z

**Published:** 2023-07-13

**Authors:** Aya Ishizaka, Michiko Koga, Taketoshi Mizutani, Ryuta Uraki, Seiya Yamayoshi, Kiyoko Iwatsuki-Horimoto, Shinya Yamamoto, Masaki Imai, Takeya Tsutsumi, Yutaka Suzuki, Yoshihiro Kawaoka, Hiroshi Yotsuyanagi

**Affiliations:** 1 Division of Infectious Diseases, Advanced Clinical Research Center, The Institute of Medical Science, The University of Tokyo, 4-6-1 Shirokanedai, Minato-ku, 108-8639 Tokyo, Japan; 2 Department of Computational Biology and Medical Sciences, Graduate School of Frontier Sciences, The University of Tokyo, 5-1-5 Kashiwanoha Kashiwa 277, 8562 Chiba, Japan; 3 Division of Virology, Department of Microbiology and Immunology, Institute of Medical Science, The University of Tokyo, Tokyo, Japan; 4 The Research Center for Global Viral Diseases, National Center for Global Health and Medicine Research Institute, Tokyo, Japan; 5 Department of Infectious Diseases and Applied Immunology, IMSUT Hospital of Institute of Medical Science, The University of Tokyo, Tokyo, Japan; 6 Department of Infectious Diseases, The University of Tokyo, Tokyo, Japan; 7 Influenza Research Institute, Department of Pathobiological Sciences, School of Veterinary Medicine, University of Wisconsin–Madison, Madison, WI USA; 8 Pandemic Preparedness, Infection and Advanced Research Center, The University of Tokyo, Tokyo, Japan

**Keywords:** SARS-CoV-2, COVID-19, Microbiota, Commensal bacteria

## Abstract

**Background:**

The mucosa serves as the first defence against pathogens and facilitates the surveillance and elimination of symbiotic bacteria by mucosal immunity. Recently, the mRNA vaccine against SARS-CoV-2 has been demonstrated to induce secretory antibodies in the oral and nasal cavities in addition to a systemic immune response. However, the mechanism of induced immune stimulation effect on mucosal immunity and commensal bacteria profile remains unclear.

**Methods:**

Here, we longitudinally analysed the changing nasal microbiota and both systemic and nasal immune response upon SARS-CoV-2 mRNA vaccination, and evaluated how mRNA vaccination influenced nasal microbiota in 18 healthy participants who had received the third BNT162b.

**Results:**

The nasal S-RBD IgG level correlated significantly with plasma IgG levels until 1 month and the levels were sustained for 3 months post-vaccination. In contrast, nasal S-RBD IgA induction peaked at 1 month, albeit slightly, and correlated only with plasma IgA, but the induction level decreased markedly at 3 months post-vaccination. 16 S rRNA sequencing of the nasal microbiota post-vaccination revealed not an overall change, but a decrease in certain opportunistic bacteria, mainly Fusobacterium. The decrease in these bacteria was more pronounced in those who exhibited nasal S-RBD IgA induction, and those with higher S-RBD IgA induction had lower relative amounts of potentially pathogenic bacteria such as Pseudomonas pre-vaccination. In addition, plasma and mucosal S-RBD IgG levels correlated with decreased commensal pathogens such as Finegoldia.

**Conclusions:**

These findings suggest that the third dose of SARS-CoV-2 mRNA vaccination induced S-RBD antibodies in the nasal mucosa and may have stimulated mucosal immunity against opportunistic bacterial pathogens. This effect, albeit probably secondary, may be considered one of the benefits of mRNA vaccination. Furthermore, our data suggest that a cooperative function of mucosal and systemic immunity in the reduction of bacteria and provides a better understanding of the symbiotic relationship between the host and bacteria in the nasal mucosa.

**Supplementary Information:**

The online version contains supplementary material available at 10.1186/s12985-023-02113-z.

## Background

Recent reports indicate that SARS-CoV-2 (severe acute respiratory syndrome coronavirus 2) infection induces a strong humoral immune response characterized by the production of virus-specific antibodies of immunoglobulin M (IgM), IgG, and IgA isotypes [1–3]. These antibody inductions are not limited to detection in plasma but have also been shown to be secreted in saliva, and a strong correlation between antibodies to SARS-CoV-2 in blood and saliva has been reported [4, 5]. Several vaccines have been developed to control SARS-CoV-2 infection, including two mRNA vaccines, BNT162b2 (Pfizer/Biotech) and mRNA-1273 (Moderna). These mRNA-based vaccines are administered intramuscularly to induce antibodies against SARS-CoV-2 spike protein, including neutralizing antibodies (NAb) against the receptor binding domain of SARS-CoV-2 (anti-S) [6].

Protective anti-S IgG and anti-S IgA have been detected in the peripheral blood and respiratory tract after post vaccination in recent reports [7–14]. In particular, IgA is secreted mainly on the mucosa as a dimer and is known to inhibit the entry of pathogens into the mucosa, and these antibodies are expected to be effective in preventing infection [15]. Some papers have reported that the amount of antibodies secreted in saliva is lower than in blood after recent SARS-CoV-2 mRNA vaccination [9, 12, 13]. It has also been reported that antibody induction by vaccination is more pronounced in previously infected individuals than in uninfected individuals [7, 8, 10–14], and post vaccination break-through infected individuals who experienced infection have been reported to have statistically significantly lower levels of anti-S IgA in saliva than uninfected individuals [13]. These reports suggest that anti-S IgA induction in the mucosa epithelium, despite its low value, may be useful in protection against certain viral infections.

The nasal and oral cavities are major entry routes for numerous respiratory viral infections [16]. The indigenous microbiota coexisting in these cavities may be altered by infection or the immune response to infection. Although there is limited information on the contribution of commensal microbiota to SARS-CoV-2 infection, reports show that the nasal and gut microbiotas are altered depending on SARS-CoV-2 infection [17–21]. Especially in the case of severe coronavirus infection 2019 (COVID-19), it has been reported that the nasal microbiota profile contributes to the spread of secondary bacterial infection (bacteremia) [22], and dysbiosis due to viral infection leads to the growth of opportunistic pathogens [23]. Although the causal relationship between the immune response to viral infection and changes in the composition of the microbiota has not yet been fully elucidated, it is suggested that the host immune response and antibody secretion in the mucosal epithelium may alter the composition of the microbiota in the nasal mucosal epithelium. Recent reports have shown that the gut microbiota and COVID-19 mRNA vaccination affect each other: the gut microbiota influences vaccine-induced immunity and changes its composition after vaccination [24–26].

Unlike systemic immunity, the immune system in the intestinal epithelium and mucosa constantly monitors and eliminates microorganisms as well as establishes a highly symbiotic relationship with the corresponding bacterial communities [27]. Secretory IgA (sIgA) is the major antibody isotype in secretions, and it monitors resident bacteria. IgG is transported through nasopharyngeal epithelial cells via the neonatal Fc receptor FcR, and regulation by these immunoglobulins is postulated to be involved in commensal microbiota diversity [27]. In addition, certain bacteria have been reported to function as adjuvants to immune acquisition in vaccination, suggesting that the intestinal microbiota plays an unknown role in promoting immunity in response to vaccination [28]. Because the mucosal environment forms the boundary between the internal and external environments and is the site of conflict between host-side immunity and symbiotic mucosal microbiota, it can be inferred that the interaction between antibodies secreted on the mucosa and the microbiota is important to maintain homeostasis [27]. However, little is known about the relationship between mucosal immunity and the nasal microbiota. Therefore, this study aimed to clarify the correlation between mucosal immunity and the nasal microbiome by examining the amount of antibodies secreted into the plasma and nasal mucosa and the changes in the nasal microbiome in participants vaccinated with mRNA against the SARS-CoV-2 S protein.

## Methods

### Subject recruitment and sample collection

Between December 2021 and June 2022, nasopharyngeal swab samples and blood samples were collected at IMSUT Hospital, the University of Tokyo Institute of Medical Science Hospital, from 18 healthy participants who are medical workers. Prior to participation in the study, all participants were negative for antibodies to the N protein of SARS-CoV-2 and had received two doses of the Pfizer-BioNTech COVID-19 mRNA vaccination (BNT162b). Participants received the third dose of BNT162b in this study, and specimens were collected before and at 1, 4, and 13 weeks after the third BNT162b. The swab was saturated in universal viral transport (Becton Dickinson, NJ, USA), and the aliquots were frozen at -20 °C until further processing. The plasma fraction of blood samples was stored at -80 °C.

### DNA extraction, amplification, and 16 S rRNA gene sequencing

We extracted bacterial genomic DNA from frozen swab samples using the DNeasy PowerSoil Pro kit (Qiagen, Hilden, Germany) according to manufacturer’s instructions. The 16 S rRNA gene libraries were prepared according to the 16 S Metagenomics Sequencing Library Preparation guide (Illumina, CA, USA). Briefly, the hypervariable V3–V4 region of the 16 S rRNA gene was amplified using specific primers: forward (5′-ACACGACGCTCTTCCGATCTCCTACGGGNGGCWGCAG-3′) and reverse (5′-GACGTGTGCTCTTCCGATCTGACTACHVGGGTATCTAATCC-3′), comprising Illumina adapter overhang nucleotide sequences (underlined) [29]. Next, adapter ligation for polymerase chain reaction (PCR) amplicons was performed using NEBNext Multiplex Oligos for Illumina (Dual Index Primers Set 1; New England Biolabs, MA, USA). Sequencing was performed using the MiSeq Reagent Kit v3 (600-cycle) with a 15% PhiX (Illumina) spike-in.

### Sequencing and statistical analyses

Sequences were quality filtered, denoised, and analyzed using Quantitative Insights Into Microbial Ecology 2 (QIIME 2 version 2019.4) as previously reported [30]. In brief, DADA2 was used to denoise the paired-end reads into amplicon sequence variants [31]. A bacterial taxonomic classification was assigned to the resulting amplicon sequence variants against the SILVA database (release 132) [32]. This was trimmed to the V3–V4 region of the 16 S rRNA gene using a naïve Bayesian classification method [33]. The Kruskal-Wallis test was used for statistical analysis of alpha diversity (Shannon index) and analyzed with QIIME2 software (cut-off p-value < 0.05). OTU tables were aligned to an equal sampling depth of 10,000 per sample by alpha-rarefaction analysis to avoid bias caused by differences in sequence depth. Data were pre-processed as described in ANCOM-II to remove low-abundance or rare taxa before differential presence ratio analysis [34]. Bacterial species identification of the microbiota was performed using the expanded human oral microbiome database (eHOMD: version 15.22) [35]. All data were statistically compared using GraphPad Prism v9. Statistical analysis of metagenomic profiles was performed using the LEfSe method to identify differentially abundant taxa.

### Antibody analysis

SARS-CoV-2 specific IgG and IgA levels were quantified using COVID-19 ELISA kits, namely IEQ-COVS1RBD-S-RBD IgA (Ray Biotech Life, GA, USA) and Anti SARS-CoV-2 S-RBD IgG ELISA Kit (Wako, Osaka, Japan) from plasma and nasopharyngeal swab. Total IgG and IgA levels were quantified using Bio-Plex Pro Human Isotyping 6-plex panel (Bio Rad, CA, USA). For comparative analysis, nasal and plasma SARS-CoV-2-specific S-RBD IgG and S-RBD IgA levels were normalized by dividing by total IgG and total IgA levels, respectively.

## Results

### Clinical characteristics of study participants

For this study, 18 healthcare workers were enrolled. All subjects enrolled in the study had received two previous doses of BNT162b2 (Pfizer/Biotech) vaccine 6 months prior to enrolment in the study, were not infected with SARS-CoV-2, and had no autologous diseases of note. Subjects received their third dose of BNT162b2 (Pfizer/Biotech) vaccine, and blood and nasal swab samples were collected at the following time points: before (Pre), 1 week (1 W), 1 month (1 M), and 3 months (3 M) after vaccination between December 2021 to June 2022. At each sample collection, quantitative RT-PCR for SARS-CoV-2 was performed and confirmed negative. The demographic and clinical characteristics of these subjects are shown in Table 1. The cohort consisted of 8 men and 10 women with a median age of 50 years (range: 25–62 years).

**Table 1:** Background characteristic of participants in this study

No.	ID	Sex^1^	Age
1	HP(H)- 007	F	45
2	HP(H)- 008	M	38
3	HP(H)- 019	F	56
4	HP(H)- 026	F	24
5	HP(H)- 065	F	40
6	HP(H)- 106	F	29
7	HP(H)- 115	F	48
8	HP(H)- 131	M	61
9	HP(H)- 168	M	49
10	HP(H)- 173	M	39
11	HP(H)- 181	M	53
12	HP(H)- 193	M	43
13	HP(H)- 194	M	49
14	HP(H)- 197	F	59
15	HP(H)- 216	F	49
16	HP(H)- 221	F	55
17	HP(H)- 303	M	50
18	HP(H)- 304	F	47

^1^ F: Female (n = 10), M: Male (n = 8)

### Humoral immunity of peripheral blood and nasal cavity to S-RBD by SARS-CoV-2 mRNA vaccination

**Fig. 1:**
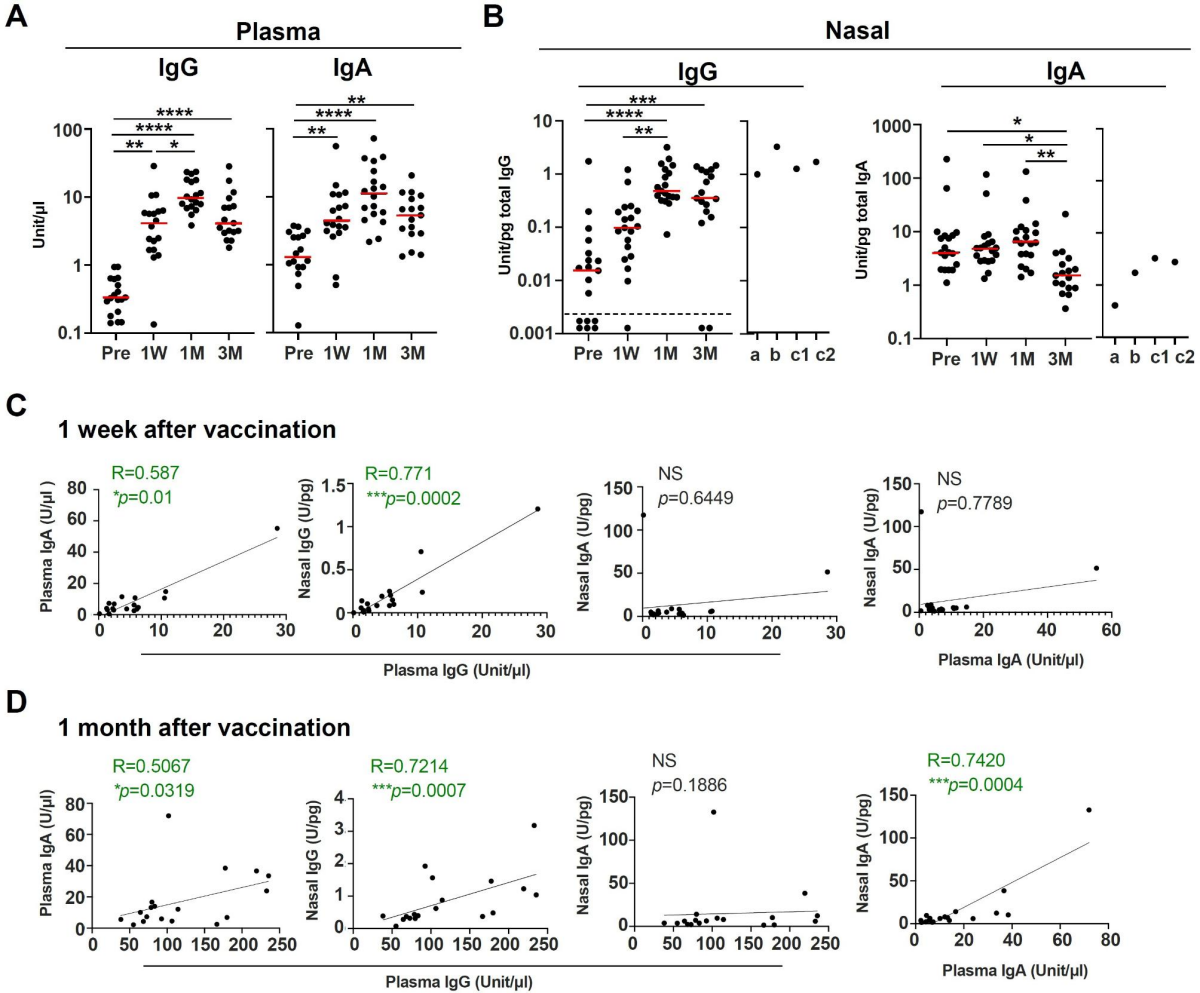
Antibody response to S-RBD of SARS-CoV-2 in plasma and nasal cavity of BNT162b2 vaccine recipients **(A)** S-RBD IgG and IgA antibodies were measured in plasma before and after the third dose of the BNT162b2 vaccination. **(B)** S-RBD IgG and IgA antibodies were measured in nasal-swab samples before and after the third dose of the BNT162b2 vaccination. S-RBD IgG and IgA of nasal swab samples from SARS-CoV-2 infected patients (n = 3). a: patient-1 (74 years old, male) at 6 days after onset, b: patient-2 (76 years old, male) at 6 days after onset and c1, c2 : patient-3 (74 years old, female) at 7 days and 28 days after onset. The dashed line represents the negative cut-offs. **(C-D)** Correlation analysis of anti-S-RBD antibodies in plasma and nasal swabs of study participants one week **(C)** and one month **(D)** after the third dose. The solid red line indicates the median for the entire subject population. *p < 0.05, **p < 0.01, *** p < 0.001 and **** p < 0.0001

Antibody responses to the receptor-binding domain on the spike of SARS-CoV-2 (S-RBD) were evaluated in serum and nasal swab samples after the third dose of BNT162b at all time-points. Nasal mucosal S-RBD IgG and S-RBD IgA were detected in nasal swabs of nearly all study participants (Supplementary Fig. 1), and antibody levels in serum were measured at the same time points for comparison. Figure 1 shows the time course of antibody levels of serum S-RBD IgG and S-RBD IgA (Fig. 1A) and nasal mucosal S-RBD IgG and S-RBD IgA of participants of this study and SARS-CoV-2 infected patients (Fig. 1B). Serum S-RBD IgG, S-RBD IgA, and nasal mucosal S-RBD IgG increased significantly from 1 W to 1 M after vaccination. Nasal mucosa S-RBD IgA increased slightly by 1 month post inoculation, although not statistically significant, but values decreased at 3 M post-vaccination. Nasal mucosa S-RBD IgG showed a statistically significant correlation with serum S-RBD IgG levels and S-RBD IgA at 1 W and 1 M after vaccination, but nasal mucosa S-RBD IgA did not correlate with them (Fig. 1C and D). However, S-RBD nasal IgA levels did not correlate with systemic S-RBD plasma IgA levels at 1 W post-vaccination, but a strong correlation was observed at 1 M post-vaccination.

### Changes in nasal bacteria abundance following mRNA vaccination

**Fig. 2:**
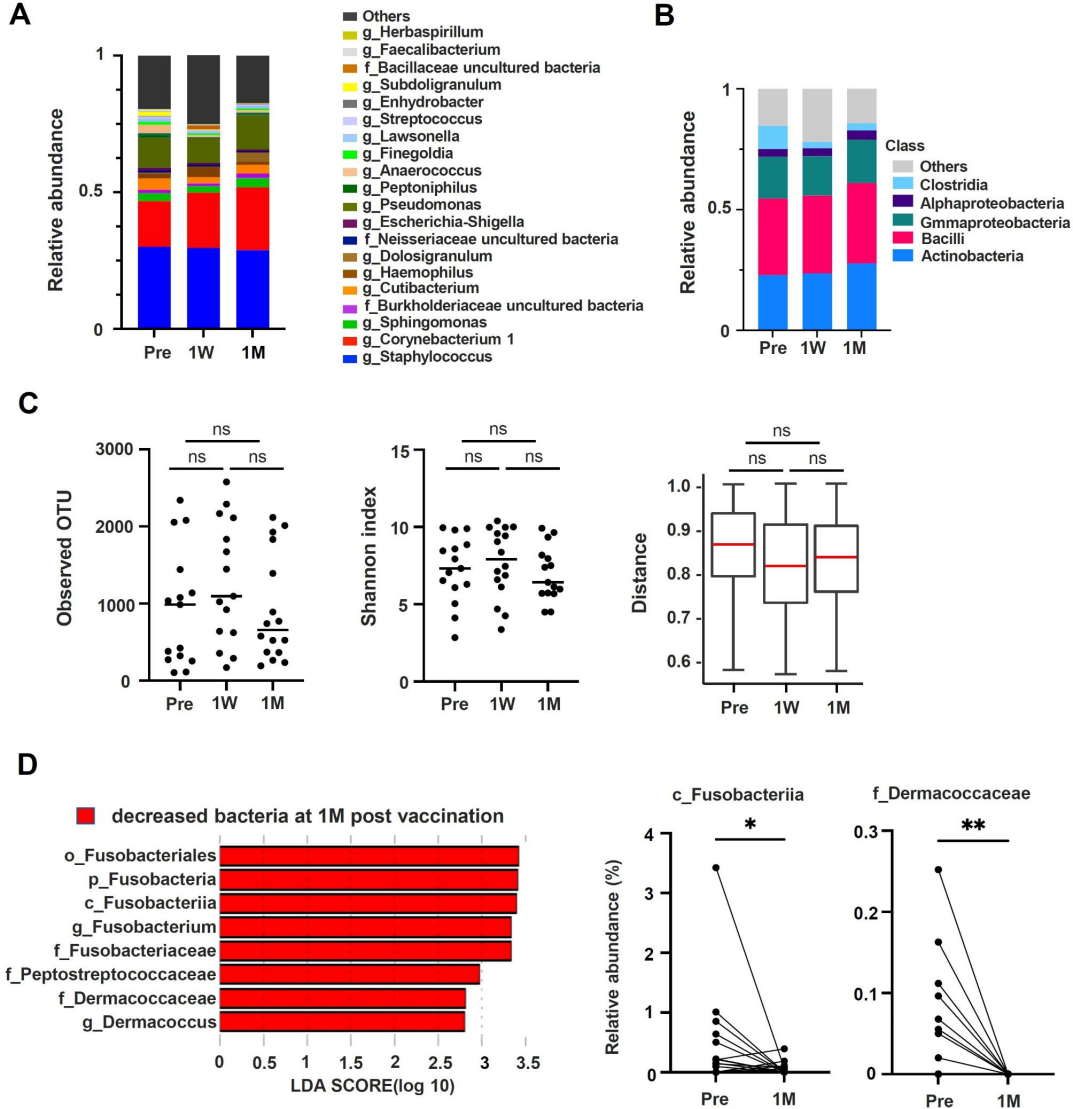
Analysis of changes to and diversity of nasal microbiota following vaccination **(A)** Taxa bar plot for main genus in the participants. **(B)** Taxa bar plot for class in the participants. **(C)** Diversity analysis of nasal microbiota in the participants. Observed operational taxonomic units (OTUs) (left), Shannon analysis (middle), and Beta diversity; unweighted UniFrac distance to pre-vaccination (right). **(D)** Changes in nasal microbiota were analyzed using linear discriminant analysis (LDA) effect size in comparison with the whole cohort (left). Changes in nasal bacteria in the nasal cavity due to mRNA vaccination. Change in relative abundance of Fusobacteriia class and Dermacoccaceae family from pre-vaccination to one-month post-vaccination (right). Wilcoxon matched pair signed rank test was used to calculate significance between pre and 1 M. ns not significant, *p < 0.05, **p < 0.01. pre: before, 1 W: 1 week, 1 M: 1 month

Next, changes in the nasal microbiota were analyzed following vaccination. After extracting DNA from the nasal swab samples, the 16 S rRNA region of bacterial origin was amplified by PCR and sequenced by a next-generation sequencer to identify the profile of the nasal microbiota. Bacterial level analysis revealed that the subjects’ nasal microbiota primarily comprised *Corynebacterium 1* and *Staphylococcus*. At the class level, the microbiota dominant in the nasal cavity were Actinobacteria, Bacilli, and Gammaproteobacteria (Fig. 2B). Diversity analysis of the nasal microbiota before and after vaccination revealed no change in either the observed operational taxonomic units (OTUs) or Shannon index nor any change in beta diversity (Fig. 2C). Next, we compared changes in nasal microbiota pre-vaccination and 1 M post-vaccination using linear discriminant analysis (LDA) effect size (LEfSe) analysis. Compared to pre-vaccination, a decrease in several microbiota was observed 1 M post-vaccination, with a decrease in the phylum Fusobacteria and its dependent bacterial species, Peptostreptococcaceae family and Dermacoccaseae family, and *Dermacoccus* (Fig. 2D, left). Among these bacteria, the reduction in abundance of Fusobacteriia class and Dermacoccaceae family at 1 M post-vaccination compared to pre-vaccination was also statistically significant in a paired t-test (Fig. 2D, right).

### mRNA vaccination induces mucosal S-RBD IgA response accompanied by changes in nasal bacteria abundance

**Fig. 3:**
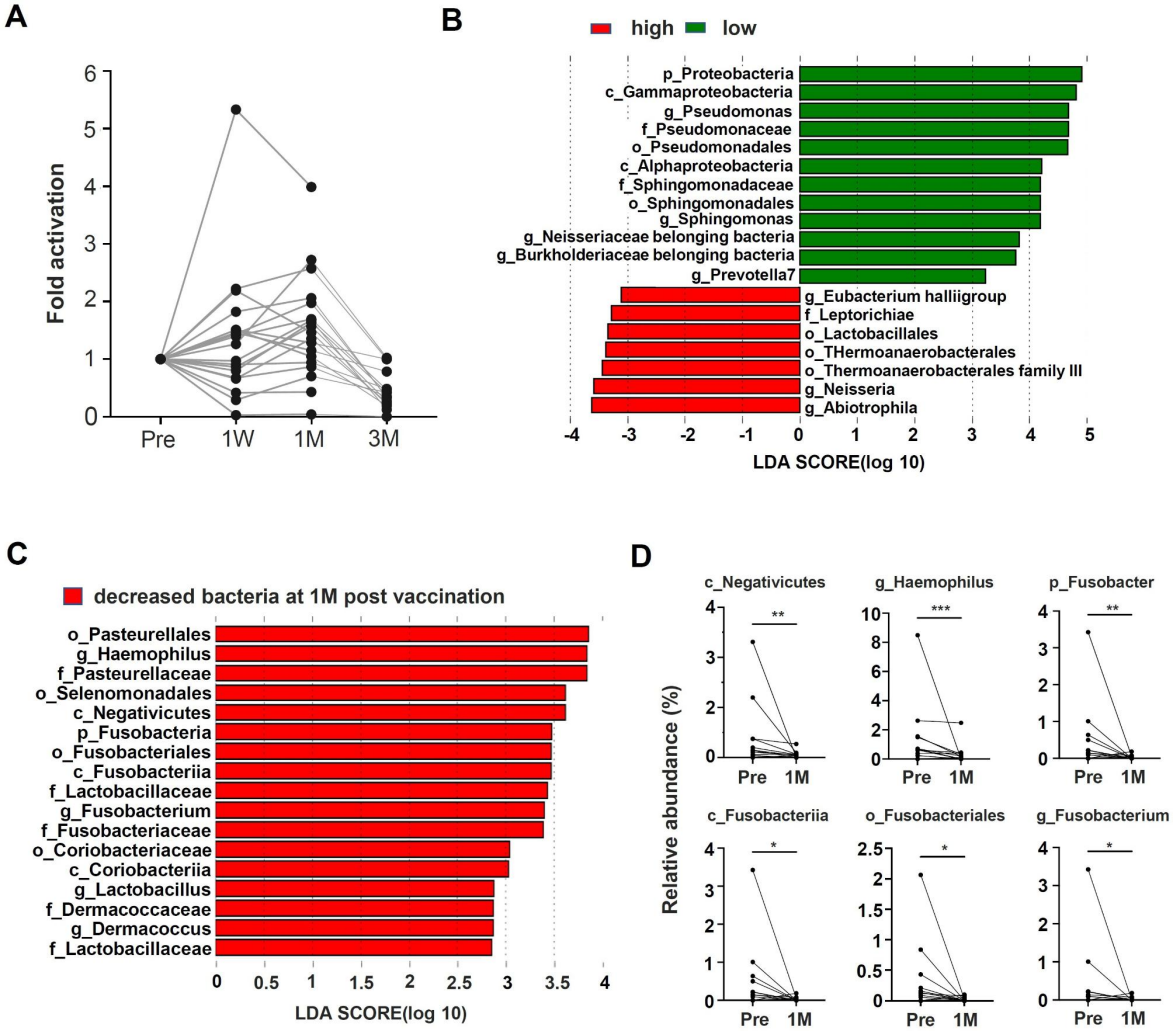
Changes in nasal IgA and nasal microbiota due to vaccination **(A)** 3-fold activation of nasal IgA level before and after vaccination. Changes in IgA values due to vaccination were calculated using the pre-vaccination value as a reference. **(B)** Values of nasal microbiota before vaccination were analyzed using LDA effect size (LEfSe) in comparison with the upper and lesser nasal IgA responders **(C)** Comparison of nasal bacterial content of IgA responders before and after vaccination (1 M) using LEfSe analysis. **(D)** Changes to bacteria in the nasal cavity in nasal IgA responders from pre-vaccination to 1 M post-vaccination. Wilcoxon matched pair signed rank test was used to calculate significance between pre and 1 M. ns not significant, *p < 0.05, **p < 0.01. pre: before, 1 W: 1 week, 1 M: 1 month

Although mucosal S-RBD IgA secreted into the nasal cavity is expected to effectively inhibit viral infection, there are few reports on the induction of nasal mucosal S-RBD IgA by mRNA vaccination against SARS-CoV-2 [36]. Therefore, we analyzed the correlation between the induction of nasal mucosal S-RBD IgA by vaccination and changes in nasal microbiota due to vaccination. Because participants in this study had already received two vaccinations, individual differences were observed in nasal mucosal S-RBD IgA levels before the third vaccination (Fig. S1). Therefore, the mucosal S-RBD IgA level at the time before vaccination was used as a baseline, and the change in nasal S-RBD IgA level due to vaccination was quantified by the rate of increase. An increase in nasal S-RBD IgA was observed in 13 of 18 subjects at 1 M post-vaccination, while no induction of S-RBD IgA was observed in the remaining 5 subjects (Fig. 3A and Fig. S1). Since it is known that secreted IgA regulates microbial diversity in the gut, we examined whether the increasing levels of S-RBD IgA affect the nasal microbiota [37–39]. Although the rate of increase in IgA within the analysis period was within a 3-fold movement in all but one sample, we selected 2-fold movement to conveniently distinguish high and low IgA induction. We then, divided the five subjects with a 2-fold or greater increase in S-RBD IgA into a high S-RBD IgA increase group (n = 5) and a lesser group into a low S-RBD IgA response group (n = 13) to examine whether values in the microbiota before vaccination affected the rate of S-RBD IgA increase by LEfSe analysis (Fig. 3B). Increased abundance of genera such as *Abiotrophila Neisseria* and *Leptotrichiaceae belonging bacteria*, as well as a decreased abundance of *Pseudomonas*, *Sphingomonas*, *Prevotella7* and bacteria belonging to the Burkholderiaceae family, were mainly observed in the high S-RBD IgA induction group compared to the low S-RBD IgA group.

Furthermore, as mucosal IgA secreted into the gut has been reported to affect microbiota [40], we compared changes in the nasal microbiota before and one month after vaccination using LEfSe analysis to evaluate the microbiota response to vaccination in 13 subjects exhibiting an increase in S-RBD IgA. The results showed that several microbiota reductions were observed 1 M after vaccination compared to that before vaccination (Fig. 3C). In particular, the reduction of *Fusobacterium* observed in the overall analysis (Fig. 2D) in addition to the Negativicutes class and *Haemophilus*, was consistently observed at each level of the paired t test (Fig. 3D). These results indicate that the abundance of certain bacterial species changed as S-RBD IgA induction occurred in the nasal mucosa.

### Association of nasal microbiota with changes in plasma S-RBD IgG levels following mRNA vaccination

**Fig. 4:**
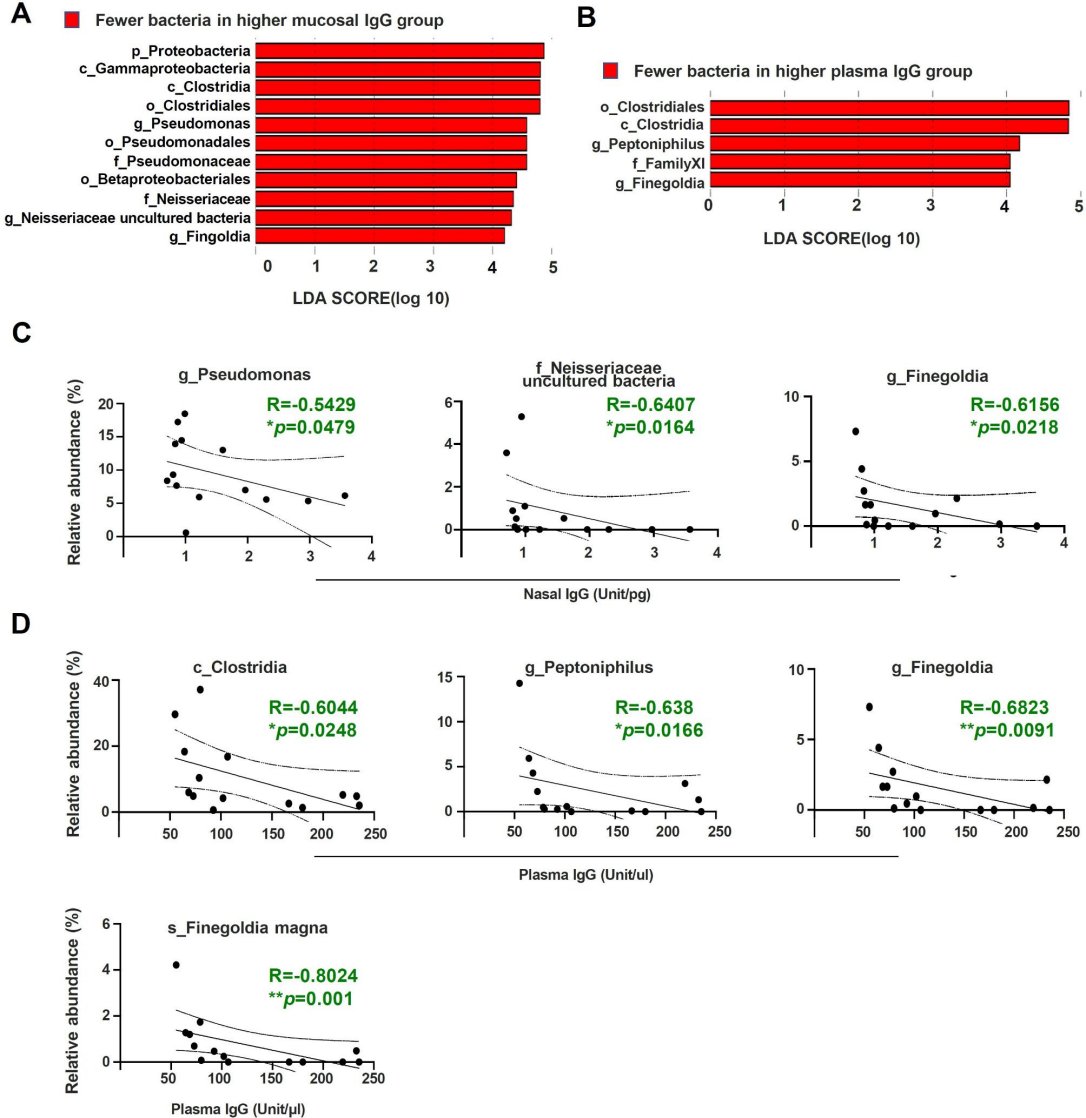
Changes in nasal and plasma IgG and alteration of nasal microbiota due to vaccination **(A-B)** Changes in nasal microbiota were analyzed using LDA effect size in comparison with the upper and lower nasal IgG responders **(A)** and plasma IgG responders **(B)**. **(C-D)** Correlation analysis between nasal IgG **(C)** or plasma IgG **(D)** post 1 month vaccination and nasal bacteria pre-vaccination. Spearman’s rank correlation test was used to calculate significance between antibodies level and relative abundance of bacteria. ns not significant, *p < 0.05, **p < 0.01. pre: before, 1 W: 1 week, 1 M: 1 month. The dotted lines represent 95% confidential intervals

The mRNA vaccine produces a viral antigen through a protein synthesis process in host cells, which triggers a strong systemic immune response. Induced S-RBD IgG is transferred to mucosal sites from the blood [27]. We analyzed the correlation between the induction of nasal S-RBD IgG and changes in the nasal microbiota caused by vaccination. Because the induction of nasal S-RBD IgG was confirmed to peak at 1 M post-vaccination, we classified the groups based on their induction levels at this time point. First, LEfSe analysis of difference between the two groups of the nasal microbiota pre-vaccination revealed that mainly *Pseudomonas*, *Finegoldia*, and bacteria belonging to the Neisseriaceae family were in smaller amounts in the high S-RBD IgA induction group than the low induction group (Fig. 4A). A similar analysis of plasma S-RBD IgG showed that the relative abundance of Clostridia, Clostridiales, Family XI, *Petoniphilus*, and *Finegoldia* decreased in the high induction group from before vaccination, as observed for mucosal S-RBD IgG (Fig. 4B). Notably, the relative pre-vaccination amounts of *Pseudomonas* as well as uncultured bacteria of the Neisseriaceae family and *Finegoldia* showed a negative correlation with mucosal S-RBD IgG levels at 1 M post-vaccination (Fig. 4C), whereas the relative pre-vaccination amounts of Clostridia class as well as *Petoniphilus* and *Finegoldia* within this class correlated negatively with plasma S-RBD IgG levels at the same time point (Fig. 4D). Further, *Finegoldia magna* exhibited a negative correlation with mucosal S-RBD IgG levels at 1 M post-vaccination (Fig. 4D).

## Discussion

Herein, our finding reveals that 3rd mRNA vaccination decreased the abundance of certain potentially pathogenic bacteria, although it did not significantly impact nasal microbiota diversity. Specifically, such decreases were associated with plasma and nasal IgA and IgG levels against SARS-CoV-2 spike and receptor binding domain (S-RBD). In this study, we observed that, compared to subjects in the low response group, subjects with higher nasal S-RBD IgA induction had lower levels of potentially pathogenic bacteria such as *Pseudomonas* and *Burkholdariaceae* before vaccination. The same group also showed reduced levels of potentially pathogenic bacteria such as Haemophilus and Fusobacterium at 1 M post vaccination.

Previous reports have shown that many bacteria in the intestinal tract are constantly coated by secretory IgA or secretory IgM, which are primarily T cell independent [27]. The function of secretory IgA is to be used for bacterial adhesion and biofilm formation, which helps increase bacterial diversity, while it is also thought to be effective in inhibiting bacterial growth and eliminating bacteria. Especially, bacterial groups recognized by IgA in normal conditions are often pathogenic and are captured and cleared by the more constrained T cell-dependent secretory IgA [37–39]. Given that secretory nasal IgA routinely monitors potentially pathogenic bacteria in the nasal mucosa to protect the host and regulate homeostasis of symbiotic bacteria, therefore, vaccination may forcibly stimulate adaptive immune response that excludes these specific bacteria in nasal cavity.

In the present analysis, the relative abundance of opportunistic bacteria, such as *Peptoniphilus* and *Finegoldia*, was lower in the group with high S-RBD IgG reactivity in the mucosa and plasma than in the group with low reactivity. Moreover, *Peptoniphilus* and *Finegoldia* showed an inverse correlation with mucosal and plasma IgG levels but not IgA, and *F. magna*, a causal pathogen of bacteremia, had a negative correlation with plasma IgG. Systemic IgG plays a role in regulating the diversity of intestinal bacteria [41–43], although the mechanism is still unclear. Nevertheless, the present observations suggest that, as in the intestinal tract, immune surveillance of the symbiotic bacterial microbiota may be functional in the nasal mucosa [44, 45]. Therefore, there is likely a coordinated but distinct bacterial-targeted regulatory mechanisms in systemic and mucosal immunity. This idea is consistent with a previous report [46].

It is unlikely that the observed changes in the microbiota directly result from immunoglobulins targeting the spike-RBD protein of SARS-CoV-2; these changes may have occurred as a secondary effect of mRNA vaccination. Thus, it is possible that the vaccination-induced transient systemic immune response enhanced a localized immune response in the nasal mucosa. The nasal S-RBD IgA and IgG levels were highest at 1 M post-vaccination and decreased at 3 M, suggesting that this secondary immune activation is less likely to continue for a long duration. At this time, it is not known if this observation will have a similar effect with different types of mRNA vaccines. However, further analysis is needed to determine the decreasing duration of the opportunistic pathogens observed in this study.

In view of the original purpose of vaccination, the observed reduction in opportunistic pathogens due to vaccination was unexpected, but this secondary effect may significantly reduce the risk of severe COVID-19. Secondary bacterial infection (bacteremia) during hospitalization is known to be a major risk factor for the severity of COVID-19 infection, and nearly 50% of hospitalized patients with COVID-19 suffered hospital-acquired infection [22]. It has been reported that opportunistic pathogens can invade the bloodstream, initiated by dysbiosis due to SARS-CoV-2 infection [23]. In particular, nosocomial infections are often caused by pathogenic bacteria that live symbiotically in the patient’s nasal cavity, and the use of antibiotics and antivirals, in addition to a weakened immune system, has been reported to cause the spread of secondary bacterial infection [22, 23, 47].

Among the bacteria decreased in this study were bacteremia-causing opportunistic pathogens, such as *Pseudomonas*, *Peptoniphilus*, and *Finegoldia*. Particularly, *Pseudomonas* and *Peptoniphilus* are more likely to be detected in the nasal passages of SARS-CoV-2-infected individuals [48, 49]. In addition, research on rhinoviruses, which cause upper respiratory tract infections, has reported that the infection severity is increased when the abundances of opportunistic pathogens such as *Pseudomonas* in the nasal cavity are high before infection [50]. These reports imply that, as is true for many infections and diseases, suppressing the growth of nasal pathogens may prevent severe COVID-19 infections. In addition, the findings of the present study suggest that the mRNA vaccine against SARS-CoV-2 may be effective in preventing the growth of these pathogenic bacteria.

Even concerning *Fusobacterium*, whose decrease was observed in this study, *F. nucleatum* belonging to this family has been reported to be highly pathogenic to humans causing periodontal disease and oral lesions [51]. Of note, an in vitro study in A549 lung cell line showed that *F. nucleatum* upregulates the expression of ACE2, a receptor for SARS-CoV-2 [52]. We are also beginning to understand the role of *F. nucleatum* in promoting inflammation in the intestinal tract. Moreover, reduced Fusobacterium in the upper respiratory tract due to vaccination may be effective in inhibiting de novo infection of SARS-CoV-2. Based on these observations, these observations propose a new aspect of vaccine effectiveness in terms of changes in the symbiotic bacterial microbiota in the nasal cavity. However, further analyses are needed to understand the role of the bacterial microbiota.

In summary, we observed a decrease in potentially pathogenic bacteria via mRNA SARS-CoV-2 vaccination in this study. However, this observation was the result of the third mRNA vaccination and a combination of factors, including the persistence of the vaccine effect up to the second vaccination, must be considered. The changes in the nasal bacterial microbiota at the time of initial vaccination and vaccination against other pathogens remain to be unraveled. Although this study was based on intramuscular injection of mRNA vaccine, differences in vaccination methods may also lead to different results. In addition, the limitation of this study is that a small number of participants and a limited cohort were analyzed. In addition, as with intestinal microbiota, the nasal microbiota has been shown to vary among patients of different ethnic backgrounds [53]; therefore, a more extensive cross-sectional analysis is required for a more robust conclusion. Although the host immune response in the nasal environment remains unknown, a comprehensive understanding of the relationship between the immune response in the nasal cavity, infectious pathogens, and nasal symbiotic bacteria is desired.

## Conclusion

The protective effect of vaccination against foreign pathogens on the nasal mucosa has focused primarily on the induction of secretory IgA antibodies so far. Given those humoral immune responses at mucosal surfaces are known to regulate symbiotic bacterial diversity, vaccination may affect the balance of commensal bacteria. However, the effects of vaccination on the commensal microbiota of the nasal mucosa are not fully understood. Here, we show that nasal commensal pathogens were significantly reduced after SARS-CoV-2 mRNA vaccination, and that their amount was inversely correlated with the induction of systemic and mucosal antibody responses. Our data suggest that a cooperative function of mucosal and systemic immunity in the reduction of bacteria. This research provides new insights into the effects of vaccination, which may lead to a better understanding of the symbiotic relationship between the host and bacteria in the nasal mucosa, as well as clues for effective mucosal vaccination strategies.

## Electronic supplementary material

Below is the link to the electronic supplementary material.

Supplemental Fig. 1. Antibody response to SARS-CoV-2 S-RDB protein in nasal swabs from vaccinated participants. Total IgA and total IgG concentrations in nasal swab samples were measured and SARS-CoV-2 S-RDB specific IgA and IgG levels were normalized based on the amount of total IgA and IgG in each nasal swab sample

## Data Availability

Data described in this study are openly available in DNA Data Bank of Japan (DDBJ) (https://ddbj.nS-RBDIgAc.jp/DRASearch; accession number: DRA015909).
