# Supplementary figures and images for: Antibody induction and immune response in nasal cavity by third dose of SARS-CoV-2 mRNA vaccination
Source: Virol J. 2023 Jul 13;20:146. doi: 10.1186/s12985-023-02113-z (PMC10339591; doi:10.1186/s12985-023-02113-z)

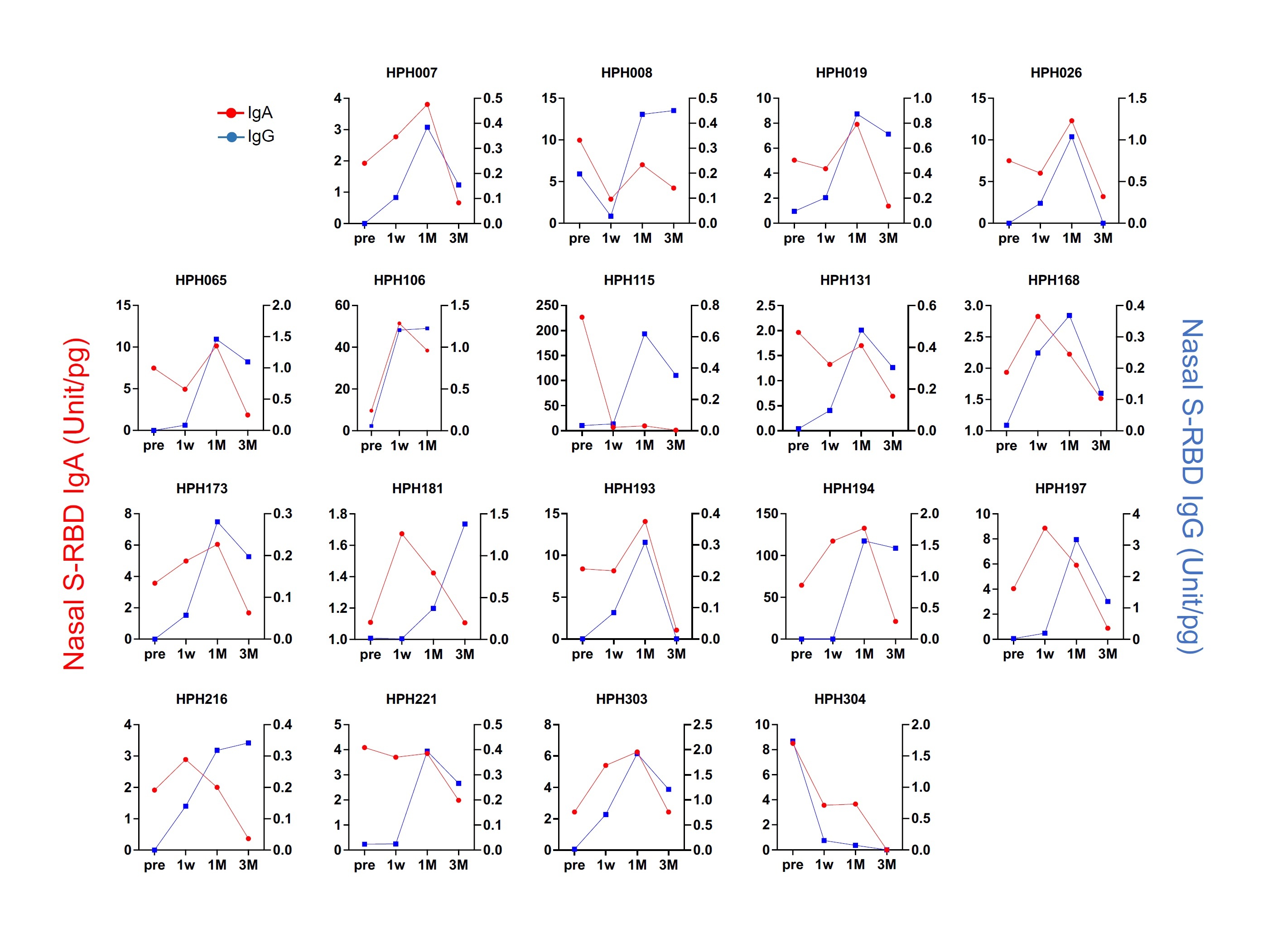

Supplement: Supplementary file 1 — Supplemental Fig. 1. Antibody response to SARS-CoV-2 S-RDB protein in nasal swabs from vaccinated participants. Total IgA and total IgG concentrations in nasal swab samples were measured and SARS-CoV-2 S-RDB specific IgA and IgG levels were normalized based on the amount of total IgA and IgG in each nasal swab sample [file 12985_2023_2113_MOESM1_ESM.jpg]
